# Efficient Photoelectrochemical Water Splitting by g-C_3_N_4_/TiO_2_ Nanotube Array Heterostructures

**DOI:** 10.1007/s40820-018-0192-6

**Published:** 2018-02-09

**Authors:** Changhai Liu, Fang Wang, Jin Zhang, Ke Wang, Yangyang Qiu, Qian Liang, Zhidong Chen

**Affiliations:** 1 School of Materials Science and Engineering, Jiangsu Collaborative Innovation Center of Photovoltaic Science and Engineering, Changzhou University, Changzhou, 213164 Jiangsu People’s Republic of China; 2 School of Petrochemical Engineering, Changzhou University, Changzhou, 213164 Jiangsu People’s Republic of China

**Keywords:** TiO_2_ nanotube arrays, Graphitic carbon nitride (g-C_3_N_4_), Heterojunction, Photoelectrochemical, Water splitting

## Abstract

Well-ordered TiO_2_ nanotube arrays (TNTAs) decorated with graphitic carbon nitride (g-C_3_N_4_) were fabricated by anodic oxidization and calcination process. First, TNTAs were prepared via the anodic oxidation of Ti foil in glycerol solution containing fluorinion and 20% deionized water. Subsequently, g-C_3_N_4_ film was hydrothermally grown on TNTAs via the hydrogen-bonded cyanuric acid melamine supramolecular complex. The results showed that g-C_3_N_4_ was successfully decorated on the TNTAs and the g-C_3_N_4_/TNTAs served as an efficient and stable photoanode for photoelectrochemical water splitting. The facile deposition method enables the fabrication of efficient and low-cost photoanodes for renewable energy applications.

**Electronic supplementary material:**

The online version of this article (10.1007/s40820-018-0192-6) contains supplementary material, which is available to authorized users.

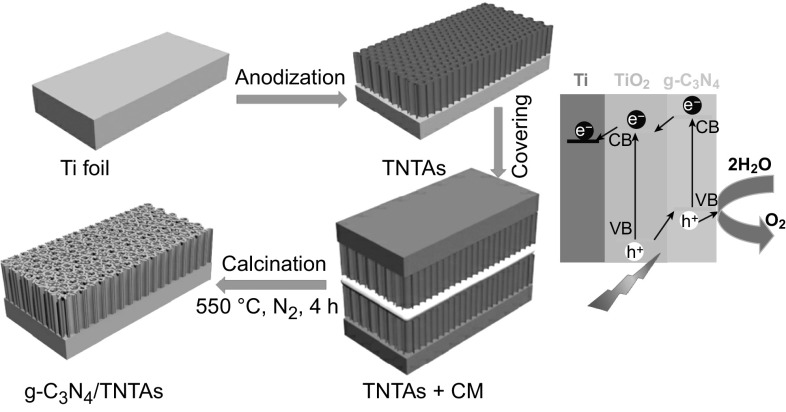

## Highlights

- Well-ordered TiO_2_ nanotube arrays (TNTAs) decorated with g-C_3_N_4_ were fabricated by anodic oxidization of titanium foil and calcination process.
- The g-C_3_N_4_/TNTA heterojunction efficiently enhanced the photoelectrochemical activity for solar light-driven water splitting. Its photocurrent density and applied bias photon-to-current efficiency were, respectively, ~ 0.86 mA cm^−2^ and ~ 0.25%, about twofold higher compared with those of pristine TiO_2_ nanotube arrays.
- The heterojunction expanded the optical absorption range of the TNTAs, accelerated the migration of carriers, and suppressed the recombination of photogenerated electron–hole pairs via an efficient band alignment between TiO_2_ and g-C_3_N_4_.

## Introduction

Photoelectrochemical (PEC) water splitting is a promising process in which solar energy is transformed into chemical energy and stored in the form of hydrogen [1–3]. In the past decades, semiconductor-based photoelectrodes, such as Fe_2_O_3_ [4, 5], CdS [6, 7], ZnO [8, 9], CuInS_2_ [10], WO_3_ [11], and TiO_2_ [12–14], for PEC cells have been extensively utilized to convert solar energy into fuel. TiO_2_ nanotube arrays (TNTAs) are commonly employed as photocatalysts owing to their excellent photochemical and chemical stability, non-toxicity, low cost, and well-aligned nanostructures. Furthermore, TNTAs have a higher specific surface area and pore volume compared to TiO_2_ nanoparticles for other active catalysts adsorbed onto the surface of both sides of nanotubes [15–17]. Despite the considerable advantages in the morphology of the one-dimensional nanostructure, the photocatalytic activity of pristine TiO_2_ is greatly limited by its wide band gap of ~ 3.2 eV, which leads to the extremely low absorption in the visible region of solar spectrum. In addition, the fast recombination rate of the photogenerated electron–hole pairs also restricts their photochemical applications. Therefore, great efforts have been made to expand its absorption range to the visible region, including decoration with precious metals [12, 18], element doping [19, 20], dye-sensitization [21], or coupling with other semiconductors to form a heterojunction [22–24].

Recently, graphite-like carbon nitride (g-C_3_N_4_), as a significant metal-free polymeric semiconductor with inherent chemical and thermal stability, and a moderate band gap of 2.7 eV, has generated a lot of interest [25–27]. Compared to transition metal oxides and sulfide semiconductor photocatalysts, g-C_3_N_4_ is composed of strong covalent bonds between carbon and nitride atoms and demonstrates high stability in acidic and alkaline electrolytes, which is favorable for PEC applications [25, 28]. However, owing to the low quantum efficiency and high electron–hole recombination rate [29], the applications of pure g-C_3_N_4_ are limited by its relatively low photoelectric conversion efficiency. Therefore, there is a significant scope to explore and design novel hybrid materials and improve the applicability of pure g-C_3_N_4_.

Herein, we fabricated a g-C_3_N_4_/TNTA heterojunction by combining anodized TNTAs on titanium foil and g-C_3_N_4_ prepared via the calcination process of the hydrogen-bonded cyanuric acid melamine (CM) supramolecular complex [30]. Owing to the existence of free hydroxyl and amine groups, the CM complex could be attached to TNTAs and amorphous TiO_2_. We have presented a facile and simple method to grow carbon nitride on the inner and outer surface of the TNTAs. More importantly, the new heterostructures of g-C_3_N_4_/TNTAs exhibit enhanced PEC water splitting activity, which is twice that of pristine TNTAs and more than four times that of amorphous TiO_2_. In addition, the results of this work proved that the heterojunctions were highly efficient as photoanodes and demonstrated stable performances for PEC water splitting.

## Experimental Section

### Chemicals

All reagents were of analytical grade and used without further purification. Ammonium fluoride (NH_4_F), cyanuric acid, melamine, and glycerol were purchased from Sinopharm Chemical Reagent Co., Ltd.

### Preparation of Photoelectrodes

**Fig. 1:**
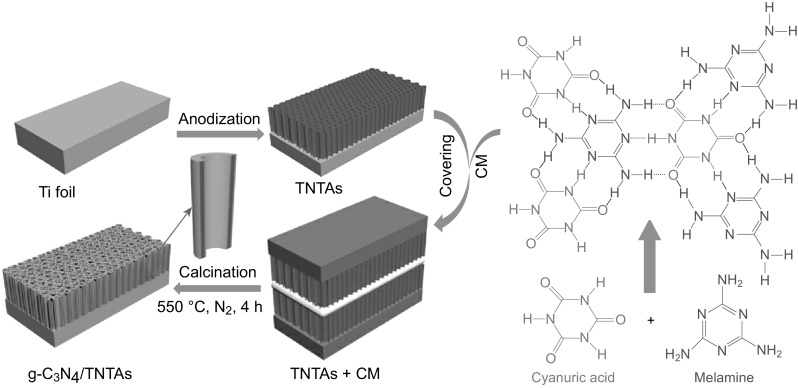
Schematic diagram of the preparation of TNTAs and g-C_3_N_4_/TNTA photoelectrodes

The preparation of the g-C_3_N_4_/TNTAs is schematically shown in Fig. 1. In a typical synthetic procedure, Ti foil (99.9%) and a platinum sheet with a size of 1 × 1 cm^2^ were used as the working and counter electrodes, respectively. The electrolyte solution was prepared by dissolving 0.5 wt% of NH_4_F in 20 mL H_2_O and 80 mL glycerol. The Ti foils (1 × 4 cm^2^ with a thickness of 0.3 mm) were cleaned by ultrasonication in acetone, ethanol, and DI water sequentially. The well-ordered TNTAs on Ti foils were synthesized via a modified one-step anodization procedure at 30 V for 2 h at room temperature. Subsequently, the obtained TNTA precursor was thoroughly rinsed with DI water, annealed at 550 °C in air at a heating rate of 3 °C min^−1^ for 2 h, and naturally cooled to room temperature.

The g-C_3_N_4_ precursor (CM complexes) was prepared by dissolving cyanuric acid and melamine (1:1) in DI water and stirring for 4 h, after which the white CM complexes were precipitated. The white powders were washed several times with DI water, and the resulting powders were dried at 60 °C in a vacuum oven. The g-C_3_N_4_/TNTA heterojunctions were prepared by calcining in a tube furnace. A specified amount of CM complexes was placed between two slices of TNTAs (or Ti). These layers were then placed in a ceramic crucible and calcined at 550 °C for 4 h under nitrogen at a heating rate of 3 °C per minute. After heating, the residual powders were blown away by a strong airstream to obtain the g-C_3_N_4_/TNTAs. In order to investigate the role of the semiconductor, two clean Ti foils without anodic oxidation treatment were used to replace the TNTAs as the substrates. After subjecting to the same calcined process, the g-C_3_N_4_ thin film was obtained on amorphous TiO_2_ and the assembly was used as a photoelectrode for comparison with g-C_3_N_4_/TNTAs.

### Structural and Optical Characterizations

The crystal structures were determined by X-ray diffraction (XRD) on a PANalytical X’Pert powder diffractometer with Cu K_α_ radiation (λ = 1.54 Å). Diffraction angle (2*θ*) ranged from 10° to 80° and the scanning step was 0.02°. The Raman spectra were obtained on a LabRAM HR Evolution spectrometer (HORIBA Jobin–Yvon) with an excitation wavelength of 532 nm. The morphology of the samples was studied by using a field emission scanning electron microscope (FE-SEM, FEI-quanta 200, 15 kV) and an atomic force microscope (AFM, Digital Instruments Nanoscope III, operating in tapping mode). The microstructure was observed by transmission electron microscopy (TEM) equipped with an energy-dispersive X-ray spectrometer (EDS). The surface compositions and elemental chemical states of the samples were examined by using an X-ray photoelectron spectrometer (XPS) with K-Alpha 1063 (Thermo Fisher Scientific, UK) instrument equipped with an Al Kα monochromator X-ray source. The light absorption of the samples was recorded with a UV–Vis spectrophotometer (UV-2500, Shimadzu, Japan).

### Photoelectrochemical Measurements

The PEC properties of the fabricated samples were measured on an electrochemical workstation (CHI660E) comprised of a three-electrode cell system of an Ag/AgCl reference electrode and a platinum counter electrode in an aqueous solution of 0.1 M Na_2_SO_4_ as the electrolyte. Light was provided by a 300-W Xe arc lamp and its power density was adjusted to 100 mW cm^−2^. In our study, all potential readings have been reported with respect to the reversible hydrogen electrode (RHE) using the equation: *E*_RHE_ = *E*_Ag/AgCl_ + (0.059 × 5.6) + 0.197 = *E*_Ag/AgCl_ + 0.53 (V). Linear sweep voltammetry (LSV) curves were collected at a scan rate of 10 mV s^−1^ with or without illumination. The periodically illuminated LSV measurement with on–off light was also recorded. In addition, the electrochemical impedance spectra (EIS) of the different photoelectrodes were obtained in the frequency range of 100 kHz–0.1 Hz without applied bias. The Mott–Schottky plots were obtained at 10 kHz frequency. The transient open-circuit potentials (OCPs) were also measured in the dark and under light illumination.

## Results and Discussion

**Fig. 2:**
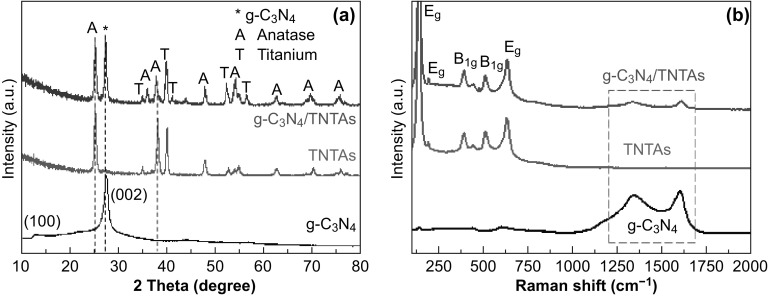
**a** XRD and **b** Raman spectra of g-C_3_N_4_, TNTAs, and g-C_3_N_4_/TNTAs

XRD studies were performed to investigate the phase purity and crystallographic structure of the as-prepared g-C_3_N_4_/TNTAs photoanode. As demonstrated in Fig. 2a, the TNTAs belong to the pure anatase phase (JCPDS card No. 21-1272) [31], and their crystal structure was almost unchanged after the loading of g-C_3_N_4_. The diffraction peaks at ~ 25° and ~ 37.8° of the TNTAs and the g-C_3_N_4_/TNTAs heterojunction were assigned to the TiO_2_ (101) and (004) peaks, respectively. Meanwhile, the peak at ~ 27.3° was attributed to the interlayer stacking of aromatic systems (002) of g-C_3_N_4_ (JCPDS card No. 87-1526) [32]. It is worth noting that the intensity of the (004) peak of g-C_3_N_4_/TNTAs was significantly reduced as compared with that of pure TNTAs, confirming the loading of g-C_3_N_4_ layers on the TNTA surface. The crystalline formation in the TNTAs and the loading of g-C_3_N_4_ was also confirmed by Raman scattering (Fig. 2b). Compared to pristine TNTAs, the specific peaks of the g-C_3_N_4_/TNTAs at 1337 and 1617 cm^−1^ were attributed to the symmetric *E*_2g_ vibration mode in the graphite-like structure and disordered *sp*^2^ micro-domains introduced by linking with N atoms [33–35], which indicated the successful loading of the graphitic C_3_N_4_ layer on the TNTAs. In addition, the annealed TNTAs and g-C_3_N_4_/TNTAs exhibited a series peaks at 146, 197, 395, 514, and 639 cm^−1^, which were characteristic of anatase TiO_2_ [36]. The Raman bands at 146, 197, and 639 cm^−1^ corresponded to the *E*_g_ mode, and two other peaks at 395 and 514 cm^−1^ were assigned to the *B*_1g_ mode. The Raman band observed at 144 cm^−1^ in the rutile phase of TiO_2_ is sharp but of weak intensity, which is significantly different from the intense and sharp band observed in the case of anatase [37].

**Fig. 3:**
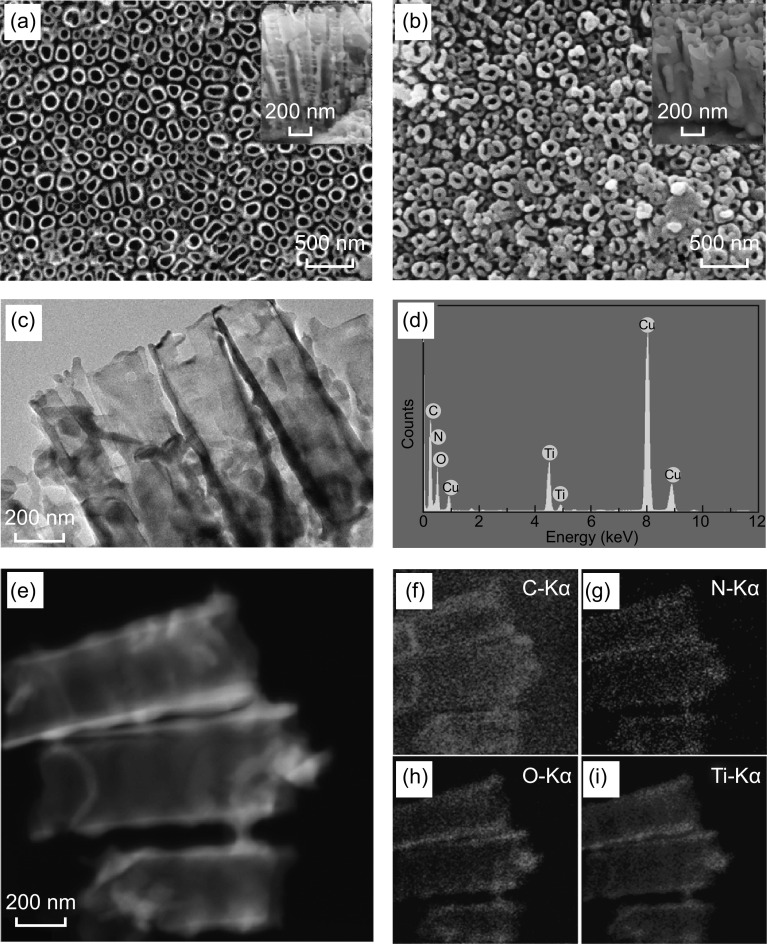
SEM images of the as-synthesized **a** TNTAs and **b** g-C_3_N_4_/TNTAs. **c** TEM image and **d** EDX spectrum of g-C_3_N_4_/TNTAs. **e**, **f** Elemental mapping results of g-C_3_N_4_/TNTAs

Figure 3a, b is the SEM images of the TNTAs and g-C_3_N_4_/TNTA layers grown on the Ti substrate, respectively. Under the optimized electrochemical anodization conditions (as described in the Experimental section), the average diameter of the TNTAs was ~ 150 nm, as shown in Fig. 3a. Subsequently, the tube layers were decorated with g-C_3_N_4_ via calcination of the CM powder under a nitrogen atmosphere. In order to compare the different roles of the tubes, titanium foil was also used as a substrate to decorate g-C_3_N_4_ under the same conditions (labeled as g-C_3_N_4_/TiO_2_). The SEM image in Fig. 3b clearly shows that after the g-C_3_N_4_ decoration process, the tube diameter decreased to only ~ 100 nm, indicating that the g-C_3_N_4_ was successfully decorated on the inner and outer walls of the tubes, leading to a decrease in the inner diameter of tubes. As described in Fig. S1, a continuous and compact g-C_3_N_4_ film was formed on the amorphous TiO_2_ surface. The TEM image of the g-C_3_N_4_/TNTAs given in Fig. 3c illustrates their distinct tubular structure; the tubes possessed a uniform diameter of ~ 100 nm. The EDS spectrum of g-C_3_N_4_/TNTAs (Fig. 3d) showed the peaks characteristic of the Ti, O, and C elements, further confirming the decoration of g-C_3_N_4_ on TiO_2_. Curiously, the nitrogen peak was not observed in the EDX spectrum. This was attributed to the low loading of g-C_3_N_4_ on the surface of the TNTAs and the low content of nitrogen in the sample. The existence of nitrogen was instead confirmed by XPS. Figure 3e shows the high-angle annular dark field (HAADF) scanning transmission electron microscopy (STEM) images of the g-C_3_N_4_/TNTAs heterojunctions. The EDS mapping analysis shows the uniform distribution of each element at the inner or outer surface of the TNTAs (Fig. 3f–i), indicating the successful coating of g-C_3_N_4_ during the high-temperature annealing process from the CM precursor.

**Fig. 4:**
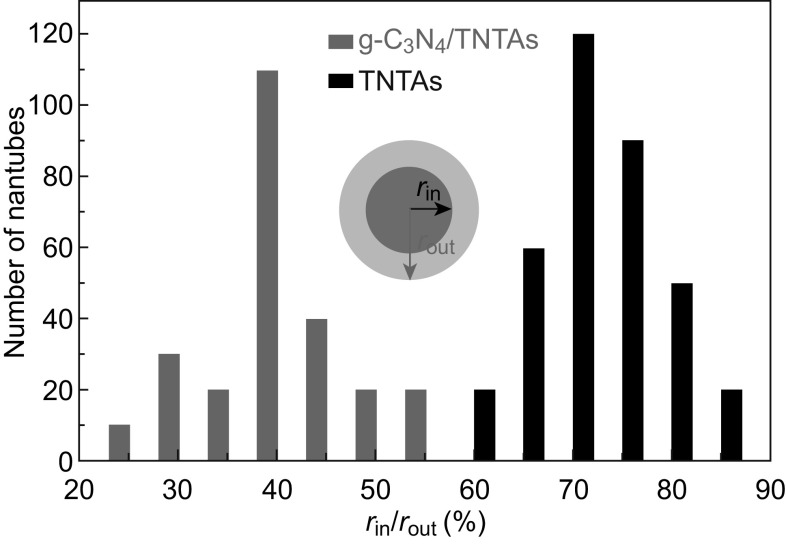
Number of nanotubes with different *r*_in_/*r*_out_ values for TNTAs (black) and g-C_3_N_4_/TNTAs (red). (Color figure online)

In order to characterize the changes in the tube diameter more intuitively, we prepared a statistical histogram of the number of nanotubes with different *r*_in_/*r*_out_ values for TNTAs and g-C_3_N_4_/TNTAs, where *r*_in_ and *r*_out_ refer to the inner and outer diameters of the nanotubes, respectively (Fig. 4). The value of *r*_in_/*r*_out_ was used to evaluate the thickness of the g-C_3_N_4_ layers, i.e., a smaller value represented a larger thickness of the g-C_3_N_4_ layers on the surface of the TNTAs and vice versa. As shown in Fig. 4, the average value of *r*_in_/*r*_out_ for g-C_3_N_4_/TNTAs was ~ 40%, which was much smaller than that of pristine TNTAs and implied that g-C_3_N_4_ was effectively decorated on the inner and outer walls of the tubes via calcination of the CM complex.

**Fig. 5:**
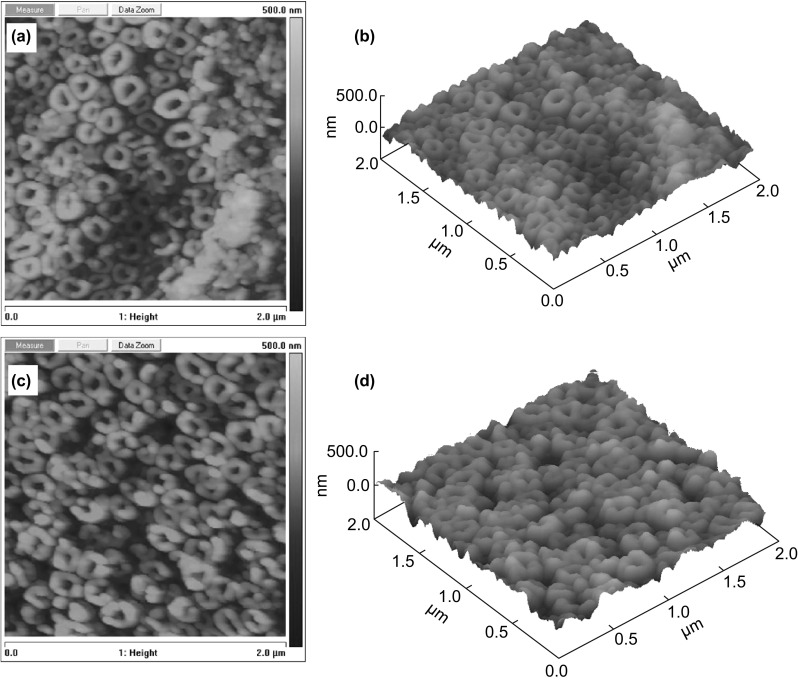
Top view and 3D AFM images (2 × 2 μm^2^) of **a**, **b** TNTAs and **c**, **d** g-C_3_N_4_/TNTAs

The surface morphology of the nanotube arrays was further investigated by AFM, as shown in Fig. 5. The characteristic two-dimensional (2D) images of the TNTAs and g-C_3_N_4_/TNTAs are shown in Fig. 5a, c, respectively. The as-prepared TNTAs with well-defined tubes can be observed, and it is also evident that the inner diameters of these tubes were greatly decreased upon coating with g-C_3_N_4_. A close inspection of the 2D AFM images revealed that the tube wall thicknesses of TNTAs and g-C_3_N_4_/TNTAs were ~ 59 and 91 nm, respectively.

**Fig. 6:**
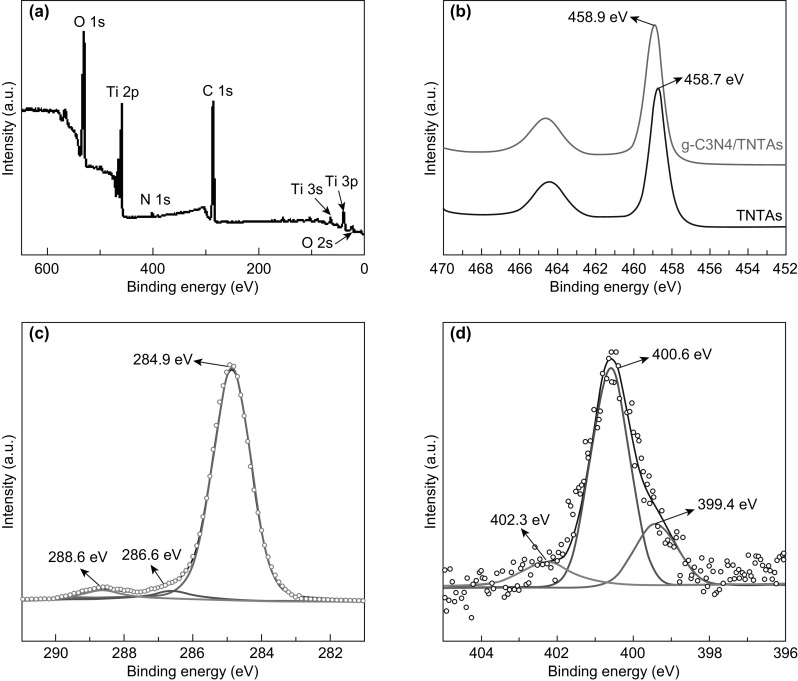
XPS spectra of g-C_3_N_4_/TNTAs: **a** survey scan, **b** Ti 2p, **c** C 1s, and **d** N 1s scans

XPS was employed to probe the surface chemical compositions and valence states of g-C_3_N_4_/TNTAs. Figure 6a presents the overall XP spectrum of the heterojunction, which indicated the presence of Ti, O, C, and a small amount of N from the g-C_3_N_4_. Figure 6b shows the high-resolution XP spectrum of the Ti 2p state in the g-C_3_N_4_/TNTAs, with the two different peaks of Ti 2*p*^1/2^ and Ti 2*p*^3/2^ and their binding energies (BE) of 458.9 and 464.6 eV, respectively, which were derived from Ti^4+^ in TiO_2_, according to the results of XRD and Raman spectroscopy. It is worth noting that the BE of Ti 2*p* in g-C_3_N_4_/TNTAs was slightly positively shifted as compared with that of pristine TNTAs, indicating the presence of interactions between the g-C_3_N_4_ and TNTAs in the form of charge transfer from the electron-rich g-C_3_N_4_ surface to the unoccupied orbital of Ti^4+^ in TiO_2_. The high-resolution C 1s XP spectrum, as shown in Fig. 6c, can be deconvoluted into three peaks at 284.9, 286.6, and 288.6 eV, indicating that carbon possesses three diverse chemical states. The peak at 284.9 eV corresponded to the signal of graphite-like *sp*^2^-hybridized C–C, ascribed to the carbon species adsorbed on the surface of g-C_3_N_4_. The peaks at 286.6 and 288.6 eV corresponded to C–OH and C–N=C bonds of the heterocycle rings, respectively [38, 39]. Figure 6d shows the XP spectrum of N 1s, which was deconvoluted into three peaks at 399.4, 400.6, and 402.3 eV. The peak at 399.4 eV was typical of the *sp*^2^-hybridized nitrogen (C–N=C), and the peaks at 400.6 and 402.3 eV corresponded to tertiary nitrogen N–(C)_3_ groups linking the structural motif and amino groups with a hydrogen atom ((C)_2_–N–H) in connection with structural defects and incomplete condensation [40–42].

To further understand the electronic band structures and photocatalytic properties, the UV–Vis diffuse reflectance spectra (DRS) were obtained to characterize the TNTAs and g-C_3_N_4_/TNTAs. Figure S2a shows the UV–Vis absorption spectra converted from the reflection absorbance spectra by the standard Kubelka–Munk method. The pristine TNTAs and g-C_3_N_4_/TNTAs exhibited an absorption edge at ~ 400 nm, and the light absorption of g-C_3_N_4_/TNTAs was greatly enhanced [43]. In order to calculate the band gaps, the corresponding (*αhυ*)^1/2^ were plotted versus the photon energy (*hυ*) [44], as shown in Fig. S2b. The band gaps of the TNTAs and g-C_3_N_4_/TNTAs were calculated to be 3.23 and 3.10 eV, respectively, by extrapolating their plots to (*αhυ*)^1/2^ = 0, according to the linear-fit lines. To determine the existence of g-C_3_N_4_ in the photoanode heterostructure, the FTIR spectrum was obtained. As shown in Fig. S3, the FTIR spectrum of pristine g-C_3_N_4_ was very similar to that of g-C_3_N_4_, consistent with previous reports [45]. The absorption peaks located at 1230, 1316, 1398, 1553, and 1631 cm^−1^ were related to the typical stretching modes of aromatic C–N [46]. The wide peak observed at wavelengths higher than 3000 cm^−1^ is usually attributed to the H_2_O molecules adsorbed on the surface of the materials. The absorption peak at 808 cm^−1^ was typical of the out-of-plane bending mode of the thiazine unit. Compared to the IR spectrum of pristine g-C_3_N_4_, a series of similar peaks were observed in the case of g-C_3_N_4_/TNTAs, which indicated a full coverage of g-C_3_N_4_ over the TNTAs.

**Fig. 7:**
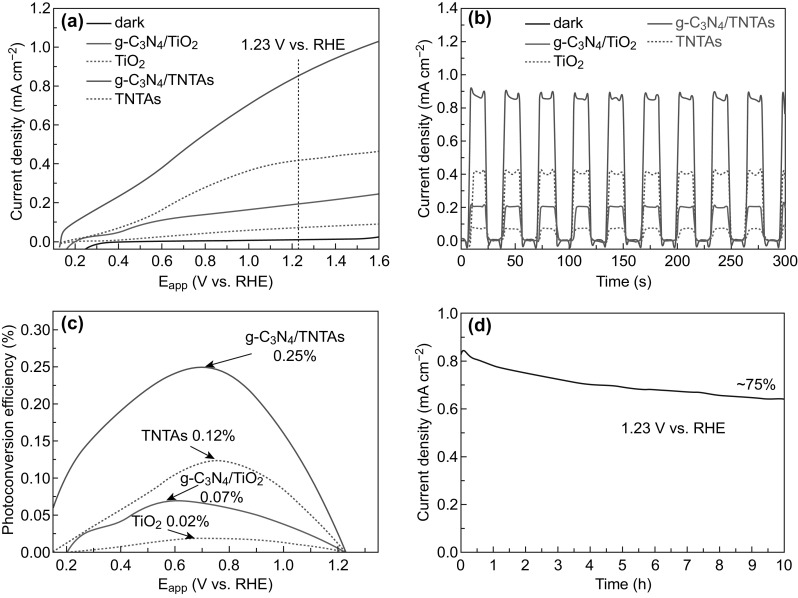
**a** Current–potential curves of the g-C_3_N_4_/TNTA photoanode and reference samples (TNTAs, amorphous TiO_2_, and g-C_3_N_4_/TiO_2_) under light irradiation (100 mW cm^−2^). The dark line indicates the dark current density. **b** Time-dependent photocurrent density of different photoanodes under intermittent light irradiation. **c** Photoconversion efficiency as a function of applied potential for different photoanodes. **d** Chronoamperometry plot (*I–t*) of the g-C_3_N_4_/TNTAs measured in 0.1 M Na_2_SO_4_ with a three-electrode system at 1.23 *V*_RHE_ for 10 h

Compared to the pristine TNTAs, the combination of TNTAs and g-C_3_N_4_ exhibits attractive features for enhanced PEC performance. A series of LSV measurements were carried out on the electrochemical workstation CHI660E. Typical plots of photocurrent density vs. bias potential in the potential window of – 0.4 to + 1.3 V versus Ag/AgCl with 0.1 M Na_2_SO_4_ (pH 6.8) as electrolyte are shown in Fig. 7a. The photocurrent density of the g-C_3_N_4_/TNTA photoanode at a potential of 0.7 V versus Ag/AgCl (i.e., 1.23 V vs. RHE) was ~ 0.86 mA cm^−2^, which was almost twice that of pristine TNTAs. Furthermore, the photocurrent densities of g-C_3_N_4_/TiO_2_ and amorphous TiO_2_ were determined to be 0.19 and 0.07 mA cm^−2^, respectively, which were obviously lower than that of the corresponding g-C_3_N_4_/TNTA and TNTA photoanodes. This suggested that the crystalline TiO_2_ nanotubes had a more distinct photoresponse in comparison with the amorphous TiO_2_ film. Figure 7b displays the transient photocurrent density (*I–t*) of the g-C_3_N_4_/TNTA and TNTA photoanodes under interrupted illumination at a potential of 0.7 V versus Ag/AgCl. With the on/off switching of light, there was a sharp increase and decrease in the photocurrent density, illustrating a quick photoresponse of the photoanodes. The photocurrents of the different photoanodes were almost of the same order of magnitude. The linear sweep voltammetry results are shown in Fig. 7a. The photocurrent density of pure g-C_3_N_4_ decorated on the surface of fluorine-doped tin oxide (FTO) glass was also tested (Fig. S4) and found to be a low value of 0.35 μA cm^−2^. This low value indicated the poor photoresponse of pure g-C_3_N_4_. Therefore, the enhanced photocurrent density of g-C_3_N_4_/TNTAs could be attributed to the synergistic effect of the heterojunction between TNTAs and g-C_3_N_4_.

The applied bias photon-to-current efficiencies (ABPE) of the photoanodes for PEC water splitting were estimated according to the following equation [47]:

1 $${\text{ABPE }}(\% ) = \, \left( {\frac{{I \times \, (1.23 - V_{\text{app}} )}}{{P_{\text{incident}} }}} \right) \times 100\%$$

where *V*_app_ is the applied external potential vs. RHE, *I* is the measured current density, and *P*_incident_ is the power density of the incident light. The calculated ABPE of the different photoanodes are illustrated in Fig. 7c. The maximum efficiency of the g-C_3_N_4_/TNTA electrode was ~ 0.25% (at 0.70 V vs. RHE), i.e., ~ 2.08 times higher than that of pristine TNTAs (0.12% at 0.76 V vs. RHE). However, the ABPE of g-C_3_N_4_/TiO_2_ and amorphous TiO_2_ were 0.07% (at 0.59 V vs. RHE) and 0.02% (at 0.72 V vs. RHE), respectively. The improved ABPE of g-C_3_N_4_/TNTAs were attributed to the creation of the heterojunction of the TiO_2_ nanotubes and g-C_3_N_4_, which could accelerate the charge migration and promote carrier separation. Furthermore, the g-C_3_N_4_/TNTAs demonstrated excellent stability (Fig. 7d), as the photocurrent remained relatively stable and retained ~ 94% of its initial value after more than 14,000 s of continuous testing under light irradiation at 1.23 V versus RHE. The outstanding PEC performance was attributed to the optimal g-C_3_N_4_/TNTAs heterojunction structure, in which the graphene-like structure of g-C_3_N_4_ enhanced photoabsorption and simultaneously accelerated the charge separation between TiO_2_ nanotubes and g-C_3_N_4_ [48].

**Fig. 8:**
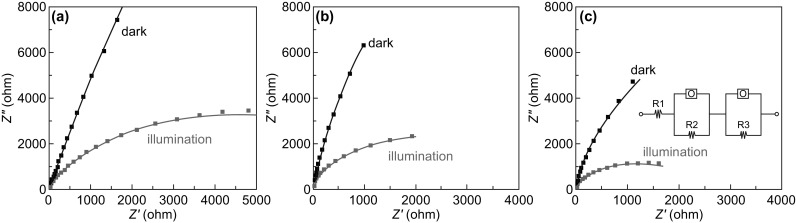
Nyquist plots of **a** g-C_3_N_4_/TiO_2_, **b** TNTAs, and **c** g-C_3_N_4_/TNTAs electrodes in dark and with light irradiation

The EIS data are an important tool obtained from the Nyquist plots to further evaluate the kinetics of charge transfer at the electrode/electrolyte interface under both dark and light irradiation conditions. The EIS Nyquist plots (Fig. 8) can be developed by the ZsimpWin software using the R((RQ)(RQ)) circuit model, including solution resistance (*R*), charge transfer resistance (*R*_3_) as the main research object, electrode resistance (*R*_2_), and electrochemical double-layer capacitance (*Q*). The fitting curve (full line) was well-matched with the experimental curve (dotted line), demonstrating a valid circuit model. It was evident that the radii for these three electrodes were significantly larger in the dark than those under light illumination, indicating a larger resistance in the former case that allowed only a few charge transmissions. Upon light irradiation, the electrode radii were noticeably reduced, among which g-C_3_N_4_/TNTAs demonstrated the largest decrease (Fig. 8c). This change indicated that the electron–hole pair separation rates and carrier migration rates were greatly enhanced in the heterojunction [49]. The specific fit values of circuit components are listed in Table 1. Under light, the *R*_3_ values for all samples were significantly reduced as compared to the resistances under darkness, among which g-C_3_N_4_/TNTAs demonstrated the largest decrease from 2.929 × 10^4^ to 2.482 × 10^3^ Ω. The significantly improved charge separation and migration potential may be responsible for the enhanced PEC performance, which is in complete agreement with the results of LSV and EIS.

**Table 1:** *Z*-fit equivalent circuit data of g-C_3_N_4_/TiO_2_, TNTAs, and g-C_3_N_4_/TNTA electrodes

	*R*(Ω)	*Q* (×10^−5^ F)	*n*	*R*_2_ (Ω)	*Q* (×10^−4^ F)	*n*	*R*_3_ (Ω)
g-C_3_N_4_/TiO_2_ (dark)	16.29	7273	0.6432	7.59	5.829	0.8858	235,500
g-C_3_N_4_/TiO_2_ (light)	17.14	4.492	1	1.649	1.156	0.8073	8928
TNTAs (dark)	7.75	1.428	1	4.516	24.66	0.9474	93,300
TNTAs (light)	11.8	1103	0.9587	1.428	3.859	0.9486	5132
g-C_3_N_4_/TNTAs (dark)	13.21	829.3	0.8474	1.569	30.16	0.9482	29,290
g-C_3_N_4_/TNTAs (light)	13.43	65.86	1	0.9062	4.037	0.9289	2482

**Fig. 9:**
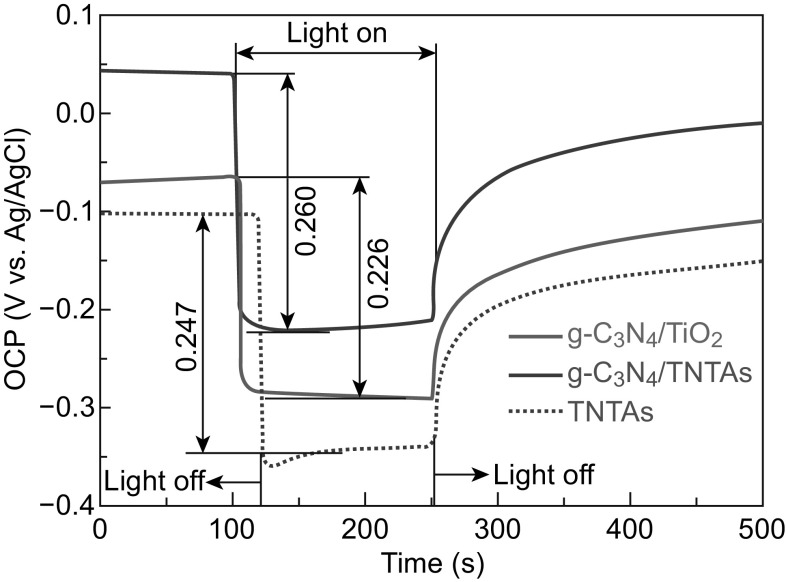
Transient OCPs of g-C_3_N_4_/TNTAs, TNTAs, and g-C_3_N_4_/TiO_2_ photoanodes

In order to explore the injection direction of photogenerated electrons, OCP transient tests of the prepared electrodes were carried out. The results of these experiments are shown in Fig. 9. All of the electrodes showed a negative increase in voltage under light irradiation, suggesting that the photogenerated electrons are injected from the semiconductor film into the Ti foil substrate [50, 51], generating the anodic photocurrent in *I*–*V* and *I*–*t* measurements. It can be inferred that the prepared films act as n-type semiconductor materials, according to the formation mechanism of anodic and cathodic photocurrent in the PEC tests. The difference between the voltages in the dark and under light illumination is the generated voltage. It is worth noting that the g-C_3_N_4_/TNTA electrode showed the largest generated photovoltage (0.260 V) among the three electrodes, which also implied its remarkable photoelectric conversion ability.

**Fig. 10:**
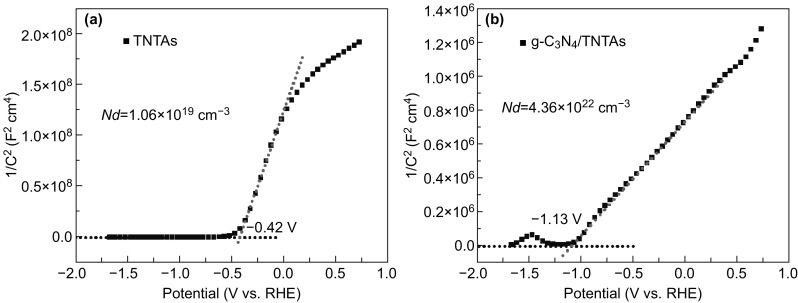
Mott–Schottky images of **a** pristine TNTAs and **b** g-C_3_N_4_/TNTAs

To investigate the charge transport behavior, Mott–Schottky (MS) measurements were performed, as shown in Fig. 10a, b, with linear segments representing the depleted states of the majority carriers in the space charge region. Both samples showed positive slopes, which implied that TNTAs and g-C_3_N_4_/TNTAs are n-type semiconductors [52]. According to the MS equation [53], the g-C_3_N_4_/TNTAs in Fig. 10b showed a much smaller slope than that of pristine TNTAs in Fig. 9a, indicating significantly higher charge carrier densities. The calculated charge carrier densities (*N*_d_) for pristine TNTAs and g-C_3_N_4_/TNTAs were 1.06 × 10^19^ and 4.36 × 10^22^ cm^−3^, respectively. Based on these values, it was inferred that the junction supplied more charge carrier density upon addition of g-C_3_N_4_. As current density is directly related to charge carrier density in a semiconductor photoanode, the calculated carrier density values strongly indicate that the enhanced PEC properties of the g-C_3_N_4_/TNTAs were a result of the increased availability of free charge carriers within the donor states of the system, which is in agreement with the PEC efficiencies (Fig. 7a, c). Overall, these conclusions can be attributed to the presence of g-C_3_N_4_ layers that promote the charge carrier separation. Moreover, the flat band potentials (*E*_fb_) of the samples were estimated by extrapolating their linear fits to 1/*C*^2^ = 0. According to the MS results, the conduction bands (CB) of pristine TNTAs and g-C_3_N_4_/TNTAs were estimated at − 0.42 and − 1.13 V versus NHE, respectively. As is well-known, the CB potential of n-type semiconductors lies close to the *E*_fb_. Therefore, the CB edges of pristine TNTAs and g-C_3_N_4_/TNTAs were at − 0.42 and − 1.13 V versus RHE, respectively.

**Fig. 11:**
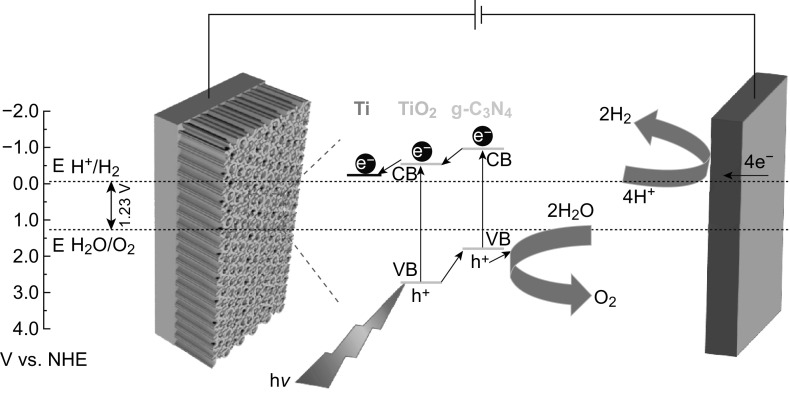
Schematic diagram of the charge transfer mechanism of the g-C_3_N_4_/TNTA heterojunction electrode

On the basis of the aforementioned results, a possible mechanism for the improvement in PEC activity over g-C_3_N_4_/TNTAs was proposed (Fig. 11). In this mechanism, a well-matched heterojunction is formed by the combination of g-C_3_N_4_ and TNTAs because the valence and conduction bands of g-C_3_N_4_ are higher than those of TiO_2_. Upon light irradiation, the electrons are excited from the valence band (VB) of g-C_3_N_4_ to its CB, which are then transferred to the CB of TiO_2_ nanotubes and leave holes in the VB of g-C_3_N_4_ [38, 54]. Finally, electrons are transported to the counter electrode through the external circuit to be consumed by H^+^ for the generation of H_2_. The direction of charge migration is in accordance with the results of Mott–Schottky and OCP measurements. In addition, the holes generated in the VB of TiO_2_ nanotubes are transferred to the VB of g-C_3_N_4_, and participate in the oxidation of water molecules. Thus, it has been established that the significant enhancement in photocurrent can be attributed to the construction of the g-C_3_N_4_/TNTAs heterojunction, which accelerates the migration of carriers and significantly suppresses the recombination of photogenerated electron–hole carriers.

## Conclusions

In summary, an efficient, easy, and universal method was used to prepare the g-C_3_N_4_/TNTAs heterojunction with a g-C_3_N_4_ shell and TiO_2_ nanotube array core, by powder coating with the cyanuric acid melamine supramolecular complex. The prepared g-C_3_N_4_/TNTAs exhibited an expanded optical absorption range and enhanced PEC activity. Moreover, the creation of a heterojunction of g-C_3_N_4_ and TNTAs significantly accelerated the migration of the charge carriers and greatly suppressed the recombination of the photogenerated electron–hole pairs. These results provide a deeper understanding of the role of semiconductor photoanodes during the PEC process of converting water to environmentally friendly hydrogen fuel.

## Electronic supplementary material

Below is the link to the electronic supplementary material.

Supplementary material 1 (PDF 606 kb)
