# Supplementary material for: Efficient Photoelectrochemical Water Splitting by g-C3N4/TiO2 Nanotube Array Heterostructures
Source: Nanomicro Lett. 2018 Feb 9;10(2):37. doi: 10.1007/s40820-018-0192-6 (PMC6199083; doi:10.1007/s40820-018-0192-6)
Supplement: Supplementary file 1 — Supplementary material 1 (PDF 606 kb) [file 40820_2018_192_MOESM1_ESM.pdf]

Supporting Information for

Efficient Photoelectrochemical Water Splitting by g-C_3N_4/TiO_2 Nanotube Array Heterostructures

Changhai Liu[1], Fang Wang[1], Jin Zhang[1], Ke Wang[1], Yangyang Qiu[1], Qian Liang[2], Zhidong Chen[2,*]

[1]School of Materials Science & Engineering, Jiangsu Collaborative Innovation Center of Photovoltaic Science and Engineering, Changzhou University, Changzhou, Jiangsu 213164, People's Republic of China

[2]School of Petrochemical Engineering, Changzhou University, Changzhou, Jiangsu 213164, People's Republic of China

*Corresponding author. E-mail: zdchen@cczu.edu.cn (Zhidong Chen)

Tel.: +86 0519-86330232; Fax.: +86 0519-86330232

Supplementary Figures

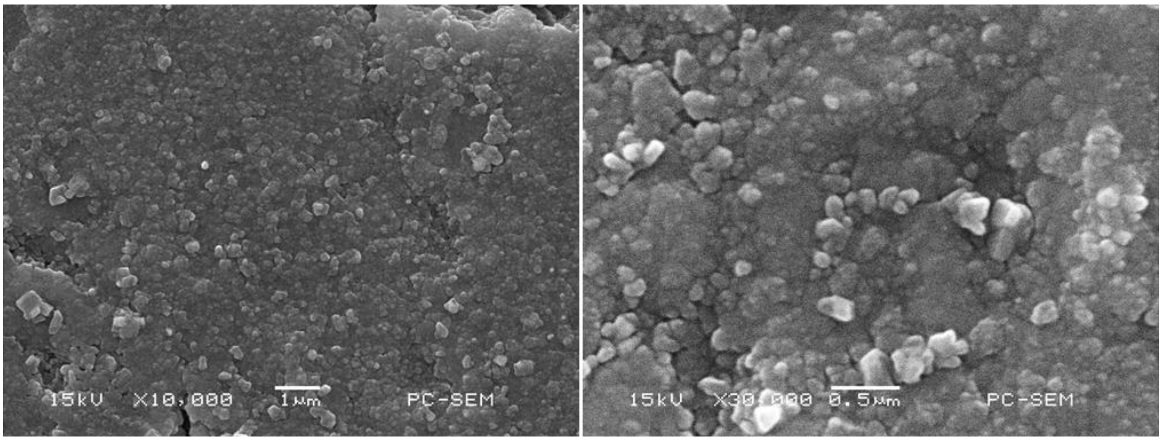

Fig. S1 SEM images of g-C_3N_4 grown on amorphous TiO_2

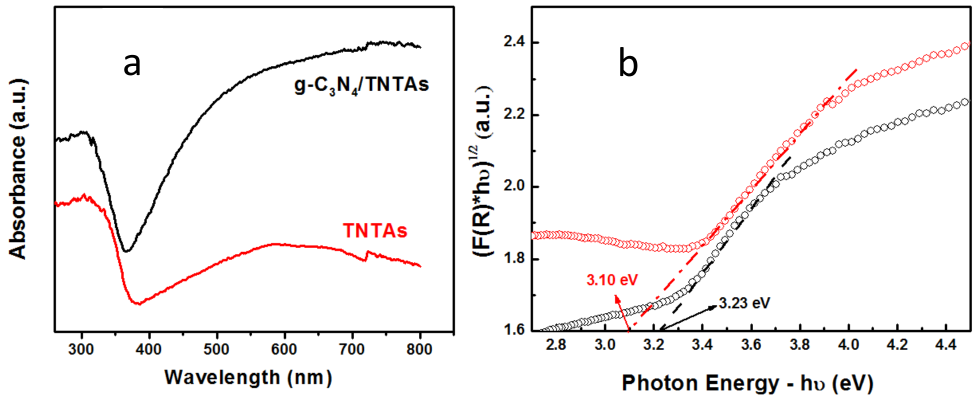

Fig. S2 a UV-vis diffuses reflectance spectra of pristine TNTAs and g-C_3N_4/TNTAs, **b** corresponding Tauc plots

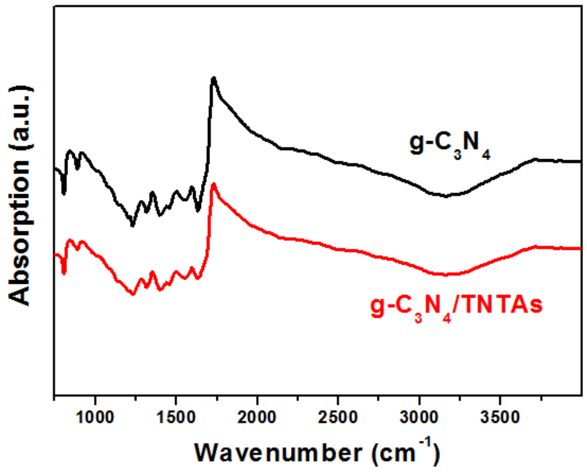

Fig. S3 FTIR spectra of pristine g-C_3N_4 and g-C_3N_4/TNTAs

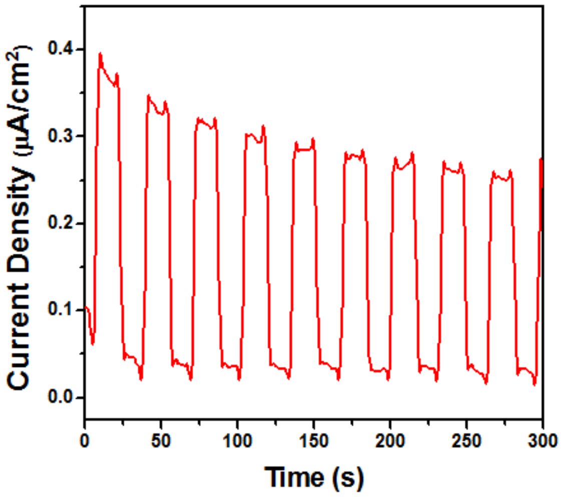

Fig. S4 Current-potential curves of pure g-C_3N_4 under light irradiation (100 mW cm^{-2})
